# The Relationship of Matter

**Published:** 1867-08

**Authors:** J. F. Sanborn


DENTAL REGISTER.

VOL. XXI.] AUGUST, 1867. [No. 8.

### Original Communications.

### THE RELATIONSHIP OF MATTER.

BY J. F. SANBORN, M. D.

*Mr. President, and Members of the Iowa State Dental Society:*

Dentistry is a specialty of that noble, learned profession, that has for its object the "cure of the ills to which flesh is heir," and not a mere trade, the routine of which may be learned in a few months.

Dentistry has been raised to its present high position, by the combined influence and self-sacrificing efforts of men of high professional attainments; exerted through Dental societies and Dental Journals, where discussions and the interchange of ideas have had an elevating influence; so that as great progress has been made in our profession as in any branch of human investigation.

The education of a Dentist, now embraces something more than a knowledge of the mechanical manipulations necessary to Dental operations; yet there is no branch of human skill that demands a higher order of mechanical ingenuity.

A knowledge of the pathological conditions that come under our observation, demands a thorough investigation of the fundamental principles of Physiology; the exigencies of the case require a reasoning from cause to effect, and from effect back to causes; and a knowledge of the relationship of remedies to disease; which in order to understand, it is necessary to investigate the essential nature of disease, and wherein lies the power of removing it.

No apology is deemed necessary for presenting for your consideration a subject not directly connected with operative manipulations; but rather with physiological principles that underlie the use of "Materia Alimentaria," as well as remedial agents, in treating pathological conditions.

In all our investigations, we are prone to take the conclusions of others as a basis from which to reason, rather than go back to first principles and investigate for ourselves.

The errors of our predecessors are continually biassing our better judgment, so that we take for our guide "incoherent, meaningless facts, without cause or use;" if the premises are false the conclusions must be wrong, and the practice based on such deductions must be defective.

On the other hand, the Godlike powers of mind when properly directed, discern the errors of the past, and reason from nature up to nature's God. A practice based on such reasoning, will have its influence in spreading the true light of science far and wide; and may such a course of reasoning guide us in all our investigations and professional operations.

Such a course has raised our noble specialty from what was once a trade, but now stands a peer among the learned professions.

Dentistry has advanced with such rapid strides, that it is only by continued exertion and investigation, that one can keep up with the improvements of the ever changing present.

Judging the future by the past, with "Excelsior" for our motto, we may hope for still higher attainments.

To do this, let us each improve the talents God has given us; let us be students ever ready to receive new ideas; always submitting them to the closest investigation of an intelligent judgment.

With these preliminary thoughts, your candid consideration is invited to our subject.

The time and space allotted for this essay are quite inadequate to do anything like justice to the subject, if it is expected that we should present it in all its phases; but rather consider our object attained, if the thoughts here presented shall induce, at least, some of the members of this society to turn their investigations to this, not new, but imperfectly explored field of thought.

All material things of which our senses can take cognizance are matter.

In our investigation we find matter presenting itself for our consideration under three forms:

1st. Inorganic matter.

2d. Organic matter.

3d. Matter in a transition state, from organic to the inorganic condition; and which may be known as broken down matter. There are certain conditions or relationship existing between these various conditions of matter, as appertaining to vitality; to which your attention is respectfully invited.

Man has a mind that allies him to God, and the matter that enters into the composition of his body relates him to earth. Mind has its influence on matter, and within certain limits, moulds it to its will.

The cranium in its form and size, accommodates itself to the brain, the seat of mentality; and not the brain to its osseous parietes.

The mind makes the man, the body is but the instrument through which the mind manifests itself; so that we should so relate the matter of which our bodies are composed, that the mind may have full scope for its highest and best attainments.

The inorganic world, is the source from which all organic matter must have its origin; and to it, it must all return. "Inorganic matter can pass to the organic, only by the vegetable kingdom, (*vide* Yeomans' Chemistry,) and in so doing it becomes organic matter, and assumes cell-form and structure; a primary form of matter, common to all organized bodies.

The breaking down of cell structure, is the first step of organized matter towards the inorganic condition; and is a change, which is continually taking place in all bodies endowed with animal life; and to replace the cell structure incident to this continual breaking down, is the demand for food.

All organized matter comes under the fiat of the Creator, "of dust thou art, and to dust shalt thou return."

Water, though inorganic matter, has its use in dissolving and carrying nutritious matter to where it is wanted, to repair and build up the tissues; and also, another equally as important a use, is to wash out, to cleanse, and purify the tissues of all broken down matter. It is a fluid prepared by God for this double purpose, and is so abundantly found in connection with the organized solids of animal life, as to constitute $\frac{7}{8}$ of the entire weight of the body, so that it is plainly seen that water bears a natural relation to the tissues of the living body; which is not the case with any of the inorganic solids.

The vegetable kingdom subsists on inorganic and broken down matter, be it animal or vegetable. The chief end of the vegetable kingdom is to elaborate matter, that is, to change inorganic into organic matter, for the use of the animal kingdom; which can appropriate organized matter, but cannot elaborate it, or raise it above the condition it is in when received.

Organic matter is found in various degrees of development; the flesh of the crustacea is very far from being as highly developed as that of the ruminatia.

The cell structure of the vital tissues, is derived from the aliment taken; so that if the food eaten is of low organization, so will the tissues, assimilated therefrom, be of a low development.

As animal life cannot elaborate matter, or raise it above the development it is in when it is received; it is obvious that it can never use matter to build up the tissues that is inorganic or broken down in its structure.

All such matter being food for plants, bears a natural relation to the vegetable kingdom, and an abnormal relation to the animal kingdom.

Webster says, "A poison is an agent capable of producing a morbid, noxious, or dangerous effect upon any thing endowed with life;" so that any thing received into the system, be it fluid, solid or gaseous, that bears an unnatural relation to vitality, adds to the amount of matter to be depurated from the system, and thereby uses up vital power, without adding to it by so doing; it occasions a morbid or noxious influence, and comes within the definition of a poison.

All matter received into the vital domain, must bear some relationship to vitality; it must be either normal or abnormal, there is no neutral ground in this relationship.

Matter in its relationship to vitality must occasion some action. What is that action? friendly or antagonistic in its character? Is that action analagous to that of matter, when according to certain fixed laws, it assumes a certain definite form, as that of the crystal? or is it like the action of matter when governed by chemical affinities? But rather, is it not an action governed by a superior power, that may well be termed vital? and is antagonistic, whenever matter that bears an abnormal relation to it, comes within its influence.

Says a distinguished author, "Life allied with matter produces combinations entirely different from those which chemical affinities of the elementary particles dispose them to assume; and preserves these combinations in opposition to their physical tendencies, as long as it continues thus associated; and this superiority is instantly shown by the readiness with which the elementary particles of that matter enters into different combinations and forms, as soon as the vital principle is withdrawn."

The vital powers by a series of changes, take matter foreign to the vital domain, and by digestion and assimilation, organizes them into the structure which it animates.

Vitality as manifested in the phenomena of vegetable life, elaborates from the crude, inorganic matter of the chaotic mass, so abundantly supplied for that purpose, from the great alembic of nature; and among other of its productions, we find the beautiful wheat berry; from which animal life can organize all the various tissues that go to make up its structure.

The doctrine of vital action, lies at the very foundation of a correct understanding of physiological principles. Organic and inorganic nature, are distinct in their essential attributes; each has its general laws peculiar to itself. "Organic matter is fundamentally distinct from the inorganic in its elementary constitution; in the aggregation of its molecules; in the structure of its parts; in its condition as a whole, and in the phenomena it evinces." Liebig says, "It is a great comprehensive law of matter, that its particles possess no self-acting, no inherent power of originating motion." All the phenomena of action as displayed in the vegetable and animal kingdom, is entirely dependent on the vital principle. In the animal kingdom there must be two classes of action.

1st. To use and appropriate matter that bears a natural relation to it, and includes all the changes embraced under alimentation and assimilation. The 2d class of actions is to remove all matter that bears an abnormal relation to it, and embraces all the changes incident to disintegration and depuration.

Health is the perfect balance in these vital changes.

Physiology teaches the phenomena of vital action, as found in health. Pathology begins where physiology ends. Unbalanced vital action is the effect, of which the abnormal relationship of matter is the cause; and disease is the effort of vitality to remove the obstructing cause, to restore the normal relationship of matter, and restore the balance in those vital changes which are incident to life.

In the relationship of matter, disease is an action; and in therapeutics it should be treated as an action to be regulated, rather than a something to be destroyed.

If disease is a vital action to remove obstructing matter, an effort of nature to restore the normal relationship, a prerequisite to health; then the use of "Materia Medica" that bears an abnormal relation, does but add to the cause of disease. Professor Paine, of the N. Y. University Medical College, in his Institutes of Medicine says, "Remedial agents are essentially morbific in their character," and "they operate as do the remote causes of disease." "We cure one disease by producing another, which we understand to mean, nature abhors the retention of matter in the system, that bears an abnormal relation to it; an action is set up to remove it; but we, by presenting a poison or matter that bears such an abnormal relation, that she abhors it more than the original cause of the disease; so she leaves her warfare on it, and directs her energies to remove the "Materia Therapeutica."

A chronic disease is almost always a drug disease, occasioned by the presence of abnormal matter, given to change or subdue the action, set up to remove the obstructing matter that has been the cause of the acute disease.

From what has been shown, it will be seen that matter in its relationship to vitality is acted on by the vital forces, and does not act of itself.

"Strong medicines" and "powerful poisons," are misnomers; they express a vital resistance to abnormal matter, and convey an impression that has no foundation in facts; for medicines do not act, but are acted upon by the "vis vitæ;" so that the true skill displayed in the administering of drugs, is to change the direction of the vital action, so as to give just enough to pass a certain point and no farther.

In our study of the relationship of matter, the laws of vitality, as we interpret them, are the use of such matter only, as bear a normal relationship to the tissues of the body; and in cases where there is disease, by following these laws we may aid nature in her efforts to cure, without causing some other disease to cure the case in hand; the acute disease will disappear on the removal of the cause that has occasioned it, and that too, without causing a chronic disease to take its place.

In practice there should be such a regard to the laws of the relationship of matter, that the remedy should not be worse than the disease.

In surgery it is often necessary to destroy the life of a part, for the good of the whole; as in the case of sphacelus cancer, or ulceration of the peridental membrane, when it would be proper to use something that would bear an abnormal relation to the part to be sacrificed, to occasion its death or removal.

In order to bring this subject more fully before your minds let us take a case in practice, and apply the foregoing principles.

Peridentitis is one of the most obscure and difficult affections we have to treat.

What is the source of the obstructing matter that is the cause of the disease? There are five outlets for depurated matter; the bowels, kidneys, lungs, liver and skin; if any one of these are not in sufficient degree of activity to perform their normal duty, some other part must perform vicarious duty, as suppressed perspiration is followed by increased activity of the kidneys or bowels, and in like manner peridentitis may be occasioned by the suppressed vital action of some other part. If so, the treatment should be directed to the removal of the general cause, and the sedative use of cold to the parts will be all that will be necessary.

A cathartic may oblige the bowels to do this vicarious duty, and thus cure one disease by producing another somewhere else, which is the "modus operandi" of "Medica Therapeutics."

The prolific cause of peridentitis is the death of the pulp, which soon becomes decomposed, the orifice for the escape of pus becomes closed by extraneous matter, and as there must be a vent for it somewhere, it is often through a foramen in the apex of the root, into the alveolus.

The pulpy cavity not properly prepared and filled, may be a cause of the disease.

If the accumulation of pus is very slight, endosmosis may remove it; but if more abundant, there will be vascular engorgement, exudation of coagulable lymph, which will become organized into a membrane for the protection of the surrounding tissues, and has been called "pyogenic membrane."

This sac becomes filled with pus, which occasions a pressure on the surrounding nerves, and pain more or less severe is the result.

The therapeutic indication in the acute variety of this disease, with much vascular engorgement, may require scarification and cold water applied locally, and a warm pediluvium, and if accompanied with fever, a warm bath is indicated, to divert the action for the removal of the obstructing matter more fully to the surface, and by so doing prevent the place of the local disease from becoming a depurating surface.

If it *does* become a depurating surface, there will be a chronic ulcer, with a discharge of pus through a fistula in the gums.

In this condition of the disease, general medication or constitutional treatment, with the hope of thus curing the local disease, is not in accordance with the laws of the relationship of matter. The case comes under surgical treatment, where the death of a part must take place for the good of the whole.

In connection with the proper observation of hygienic rules, an escarotic that shall occasion a death of the immediate surrounding vital parts, and thus destroy and break up the bad habit of depurating. This can be done with nit. arg. 60 grs. to the oz. of water, or by a saturated solution of iodine in creosote.

Either of these injected through the fistula, or pumped through the apex of the root, by means of fine prepared cotton closely wound around a Swiss broach. It requires care and skill to make the application equal to the theory; and on this depends most if not all the chances of success. In some cases it may require but two or three applications; other cases may take weeks to effect a cure.

As soon as it is proper, fill the root, after carefully wiping it out with a pledget of cotton; and according to the best Dental authority it should be slightly moistened with creosote, which it is said, forms an insoluble compound with the fluids of the parts.

The crown should be filled at some other sitting, if gold or tin is used for a plug, as there is danger of exciting a renewed attack of inflammation, if too much manipulating is done at one sitting.

In treating all the diseases to which "flesh is heir," see that the relations of matter shall always bear a natural relationship to vitality, when to save life is the desired end.

But in surgical cases, when to save life it is necessary to cause local death, then and then alone, use matter that bears an abnormal relationship to vitality.

Nature's law of cure, is true obedience to physiological law.

